# Sixteenth Annual Meeting of the Mississippi Valley Aassociation

**Published:** 1860-04

**Authors:** 


### SIXTEENTH ANNUAL MEETING OF THE MISS. VALLEY ASSOCIATION.

### DISCUSSIONS.

### I. Mechanical Dentistry.

1. *Continuous Gum Work.*

Dr. Atkinson said he had not been successful with continuous gum work. It was very pretty, and gave the operator great control as to shape and position of the teeth; but he thought it was not strong enough. If platinum were as stiff as gold plate, he thought it would answer. If it would stand, he would regard it as A No. 1; but he did not know of a case that had not to be re-made or set aside.

Dr. Richardson remarked that when built out much it was sometimes too heavy. Most of the properties of this work, he said, are already well recognized. It was very *pure*—both the metal and the porcelain; and nature could be imitated to any extent. It had not as extended a range of application as gold, and some other styles of work. When the case would admit of a sufficient quantity of body, he thought it was strong enough. But strength might be gained by making a double plate, having the two lamina separated in the palatal region the depth of an ordinary air chamber, and then filling in gum body between the plates, so as to convert the Cleveland chamber, thus formed, to Gilbert's. If the lingual surface of the plate was not enameled, this construction presented a smoother surface to the tongue.

Dr. Johnston said his experience and observation did not correspond with Dr. Atkinson's. He had not been troubled with breakage. One of the first cases he ever made had been worn seven years, and was still serviceable. The materials have been improved; and he was capable of doing better work than when less experienced.

Dr. ATKINSON thought that, to have a continuous gum job last seven years, the articulation must be very accurate.

Dr. TAYLOR said he had lately repaired one of Dr. Allen's first pieces, which was still "wearable," but very much cracked. As to his own cases, for the past ten years, a large majority have had to be made over again, or replaced with other work, but some are still standing. Notwithstanding these failures, he would not be willing to do without this style; and he regarded it as the greatest improvement in mechanical dentistry. He remarked that it was once maintained by Dr. Allen's opponents that the body would not adhere to the plate. Now the same persons cover the entire plate, and it sticks. Some people, he said, would break their teeth under any circumstances, while others seldom did. With the latter, the teeth will stay as long as the mouth remains unchanged in shape. But the mouth gradually *changes;* and as this work can not yield, by the plate pressing up, the gum body cracks. He referred to a case in which not only continuous gum, but even gold plates would break, from this cause.

Dr. ULREY said he had very little experience in the matter. Three months ago he broke up a piece which had been worn seven years. It was badly cracked. He supplied its place with gold.

Dr. JOHNSTON stated that, when preparing this kind of work, he gives it his undivided *personal* attention; and his success had been as satisfactory as with other styles of work. Its great liability to break by a fall, and the difficulty of mending it were his strongest objections to it.

Dr. TAFT said that his late experience differed very much from that of some others. Six years ago he had partially failed with this work, but he had "posted up," and the materials had been very much improved. For twenty months past he had been very successful in its use. It was not more liable to accident while making than other work, nor more so while wearing, unless it meets with a fall. Then it is more likely to break. If work fits badly, and does not antagonize properly, it will break, let it be what style it may. By annealing at a high heat, a platinum plate could be swaged to fit better than a gold one; and this was an important consideration. The plate should rest firmly on the ridge; and it might be strengthened, as described by Dr. Richardson, or by making depressions over the rugæ deeper; and also a continuous backing. For this latter mode, the single rivet teeth are preferable. The antagonism must be perfect—not striking at only one or two points. If these points were all observed, he thought it would last as long as gold work usually does. It had, also, many advantages over gold work. It is not offensive; for when the body is good, and well enameled, he maintained that it does not absorb the fluids of the mouth. He thought it as easily repaired as gold work. But this was not the question at issue; for, if it is *better*, the trouble, time or skill should not be taken into consideration. He had used it in partial sets of every variety, and has not, in the last twenty months, had to repair this oftener, in proportion, than other styles of work. Most of his temporary sets are now mounted in this style. He takes an old platinum plate, sometimes, and mills it down a little thinner than ordinary—swages and fits it, adjusts the teeth as usual for temporary work, and adapts a narrow rim of gum body to the necks of the teeth, the edge of the plate and the inequalities of the gum, and bakes and finishes in the usual method. When thus done, temporary work presents as fine an appearance as permanent. This work, he said, could not be slighted with impunity. Every step had to be perfect. With beginners, it took more time than gold work; but with equal familiarity, it did not. He now burnt it on a platinum wire frame; and, being thus uniformly heated, it seldom changes—

was not as liable to warp as gold work. When it did change, it was to be corrected by forcing it back on the cast.

Dr. RICHARDSON said that in nearly all the cases he had repaired, the teeth, and not the body, were broken. Thought continuous gum work three times as liable to break as gold work. The body formerly used, he said, was not reliable. He had not much confidence in lining the teeth so as to strengthen the work,—thought the body was strong enough. He regarded the covering of the lingual surface of the plate as a great improvement. He had not regarded the want of strength as an objection to this style of work. He thought continuous lining best—had seen cases break, he thought for want of it. He regarded this style as mainly adapted to full sets. When the under jaw was large, strong, and prominent, he thought this work would not stand well. For the last eighteen months, his success had been much better with it than formerly; he considered the new gum quite an improvement on the old.

Dr. LUPTON said he had formerly used this style of work, and abandoned it, but was now using it with more satisfaction. He referred to a partial set he had lately seen, which showed superior workmanship.

Dr. JOHNSTON said that if he had to adopt one style of work exclusively, he would take gold work with single gum teeth.

2. *Vulcanite Base.*

Dr. ATKINSON said he was slow to adopt vulcanite base, but now thought it the best material in use, especially for lower grinders when the front teeth remain. The vulcanite meets that case exactly. He exhibited a piece composed of vulcanite plate with two molars on each side, with which the patient eat hard food immediately after its insertion, without in the least displacing it. With a perfect impression, we are sure of a perfect fit. As claimed for continuous gum work, every thing must be done perfectly, and success is in proportion to the pains taken. When blocks are used with this work, he suggested that the canines be separate, that they may be placed sufficiently prominent to give the arch the natural curve. After the teeth are ground in place, he suggested the cutting of a dovetail groove in the sides of them to give the material a firmer hold. He thought the vulcanite was more pleasant in the mouth than the metal or porcelain plates; and it gave perfect control as to "plumpers."

Dr. Foote liked the vulcanite better than any other material for partial sets. For taking impressions for partial pieces, he preferred gutta percha. This should be boiled and worked till perfectly softened; then the surface should be chilled in cold water, to prevent its burning the mouth. The plaster should be *immediately* put in the impression. By covering the plaster model with tin foil before packing, he said the surface of the base is improved. The tin may be dissolved off with hydrochloric acid. He considers the work sufficiently strong, and thinks a gold plate would bend before this breaks. He had never seen a tooth come off when "pins" were used. Dovetailing alone would not quite answer the purpose. Pin teeth, he said, were stronger than others; and he would always advise the selection of strong teeth. This work, he remarked, can be mended two or three times, but by each vulcanization it is rendered darker in color and more brittle. He had not seen any difficulty with lower sets, from its lightness; and he had yet to see the first piece of rubber work break. He charged the same for it as for gold work, but not as much as for continuous gum.

Dr. Atkinson remarked that when vulcanized at too low a temperature, it was leathery; at too high a heat it was brittle.

Dr. Foote said that when the temperature was too low, the work would taste and smell of sulphur, and turn black in the mouth; but when properly vulcanized it would not. The "American Hard Rubber Company" he said, directed a temperature of 310 degrees for three hours; but this would render the substance rather brittle.

Dr. Atkinson stated that it begins to vulcanize at 270 degrees; and, by giving time enough, that temperature would do. He thought the "three hour gum" would be vulcanized by a temperature of 290 degrees, for four hours.

Dr. Richardson said he liked the work, but would suggest a difficulty which was rather annoying, especially in partial pieces. The thing is to get it out exactly as it goes in. In general, he said, there must be a superflous quantity of gum material. This would press out and prevent the moulds from coming accurately together. (Dr. Atkinson said that grooves should be made for the surplus to run out by). Dr. R., resuming, said that the excess would not always follow the grooves; and that when the moulds did not come accurately together the plate was too thick. By this means the teeth were elongated, and their gums were, in partial sets, thrown off the natural gums. A remedy had suggested itself to him, and that is a deep groove around the matrix, as close to it as practicable, with gateways leading into it. He named, as another objection, that it insinuates itself between the teeth, and gives an unsightly appearance. To prevent this he had inserted tin foil between the teeth, and dissolved it out with hydrochloric acid, after vulcanizing. He likes the vulcanite better than gold work; and regarded as not the least of its good properties, that it could be built out to any extent, without becoming too heavy. He regarded lightness as an important property, and thought their weight was an objection to continuous gum work, and cheoplasty. He had tried it in a case in which gold, silver, and cheoplasty had proved unsatisfactory, and was successful.

Dr. Foote: to prevent the surplus gum keeping the moulds apart, digs a channel around—not too close to the teeth, and makes many small gateways into it,—these gateways widening as they go from the teeth. He puts tin foil around the teeth on the plaster model, against which the gum is to be fitted. In answer to a question of Dr. H. A. Smith, he stated that, if the lip would be rendered too prominent by the gum coming over the ridge, he would grind the teeth to rest on the natural gum, and rely solely on the pins to hold them in place.

Mr. J. T. TOLAND exhibited an upper set of teeth mounted on "amber base," said to be vulcanized at 300 degrees, in four hours. It presented a neat appearance, and much more nearly approximated a gum color than the rubber specimens on exhibition.

Dr. TAFT said he liked the vulcanite about as well as he did a year ago. As to durability, he thought it would prove satisfactory—would do about as well as any thing else. Dr. Bonsall's case, made in the association a year ago, presents now a little roughness. He had noticed the same in other cases. He thought the manipulations would still be much improved. He referred to the great improvements in vulcanizers, and remarked that the boxes, or cups, must also be greatly improved. The "guides" should admit of no lateral motion; for when they do, the teeth are likely to be displaced. The boxes should be improved; and only the best plaster should be used for moulds.

Dr. RICHARDSON remarked that the guides should be longer, and the clamps should be larger, so that they will go on the flasks, before their parts are brought close together.

3. *Cheoplasty.*

Dr. H. R. SMITH said that two years ago all was alive with cheoplasty. Now the brethren seemed to be down on it, but he was going to try it. It had been highly recommended by members of the profession now present. Those who now advocate "rubber," once praised cheoplasty; and he had more faith in it now than in vulcanite.

Dr. TAFT said he liked the cheoplastic work now as well as he ever did; and he presumed that it is now as extensively used by the profession as it ever was. It can be made in less time than rubber work, and would fit equally as well. He expected to use it in some cases, and found the metal very convenient, sometimes, in making apparatus to correct irregularity. In a large majority of cases he preferred other styles of work.

Dr. ATKINSON thought that cheoplastic work would have been in better repute if it had, in general, been better made.

4. *Gold Work.*

Dr. ATKINSON remarked that the principal difficulty with gold work, when the operator is competent, is that the shape of the mouth constantly changes. The extent and rapidity of change differ in different constitutions. He cited the case of a professional man who, on this account, had found it necessary to have his work re-made, eight or ten times, in twelve or fifteen years. This, he said, constitutes the only difficulty with this style of work which is not already well understood by the profession. The causes of this difficulty would lead into Histology, and take up more time than could be devoted to it now.

Dr. H. R. SMITH hoped some one would say something in regard to the quality of the gold, and the proper thickness of plates.

Dr. RICHARDSON thought the gold should not be reduced below 20 carats. If the mouth were healthy and would always remain so, 18, or even 16 carat gold might be worn without serious change; but he thought it should be borne in mind that even the most healthy are liable to sickness, and, consequently, to a change of the character of the fluids of the mouth. In some mouths, he said, American coin would tarnish. For such, he suggested the reduction of pure gold with platinum. A quantity of platinum, sufficient to give the requisite stiffness, could be added to gold without materially injuring the color of the plate. He spoke of the qualities of solders to be used on different specimens of plate. He uses a minute quantity of zinc in gold solders. In answer to an inquiry, he stated his doubts about the zinc being all burnt out in soldering, as claimed by some.

Dr. WOODWARD stated that when a gold plate springs, from excess of solder, he cuts away the excess, and makes a cove and die of lead and bismuth, and swages it down by a succession of light taps. But, he thought when plates were properly managed they were not likely to spring.

Dr. Richardson thought springing might result from a variety of causes, such as excess of solder, irregular heating, bad investing. The change, he said, was mostly on the sloping surfaces, inside of the teeth. Here, he said, is the weak point; and as the plate expands, while the plaster contracts, with rise of temperature, these have to bear the strain. The plaster should be mixed with sand and asbestos; and as little of the mixture should be used in investing as will hold the teeth in place; and it should be nearly disconnected over the palatine portion of the plate. The work should be heated uniformly; and a broad flame should be used till the solder begins to flow. When in a hurry, he usually puts the work in hot water, as soon as it is cooled below a red heat, and he had never broken a tooth by doing so.

Dr. Atkinson said that, when a tooth breaks by too rapid cooling, the cracks will be fine, and run in various directions —by too rapid heating, it usually breaks in a single direction.

Dr. Baxter has found but little trouble from too rapid heating. When he waits till his plaster investment is perfectly dry, his plate usually warps,—when he heats it right up, it does not. He solders the piece with lamp and blowpipe, without taking it from the furnace.

### II. What is the best treatment when the pulp of a tooth is exposed, in order to secure its vitality?

Dr. Baxter said that, in recent exposure, if the pulp bleeds, he lets it bleed till it ceases of itself, and then fills with gold—sometimes inserting a little asbestos, as a non-conductor. He thinks that after the flow of red globules ceases, serum exudes, and renders the use of escharotics useless.

Dr. Focte was glad to hear that the gentleman avoids irritating the nerve. In slight and recent exposure, he would put in prepared flax and fill over it with Hill's stopping. After a while he would remove this, and fill with gold. Should there be soreness of the tooth afterward, he would use electricity. This, however, should not be applied to an irritable or inflamed surface. To illustrate,—If the elbow was inflamed, he would pass the current down the forearm, to the hand, but not through the elbow. To relieve tooth-ache, he would put the negative pole to the feet, and the positive to the upper part of the spine, and adjacent parts. The effect of an electric current, he said, was to increase the circulation; and this fact he regarded as important. He would relieve violent headache by the mode above described for toothache, and would even treat inflammation of the jaw on the same general plan. In none of these cases would he use a strong current.

Dr. McClelland said that when the pulp has been exposed for several days he does not expect to save it alive. When the fluids of the mouth had reached it, he did not think its vitality could be preserved by treatment, as he believed effusion would take place. To ascertain whether the pulp is exposed or not, he directs the patient to suck the tooth, which will produce pain in cases of exposure. In cases of accidental exposure, while excavating, he would wait till the hemorrhage abated, then wash out the cavity with tepid water, and fill with Hill's stopping. Sometimes he applied a little tannin. He was not very partial to treating nerves, though he had tried creosote lately. When escharotics are used, he thinks a dead portion must slough off, and that there will be effusion in consequence of this. His temporary fillings, as described above, he allows to remain some six months, before filling with gold.

Dr. Driggs inquired how it was to be known that exposed pulps remained alive after filling.

Dr. McClelland said he had found the dentine normally sensitive, when he removed his temporary, to insert permanent fillings.

Dr. BAXTER said he had seen a case in which the nerves were alive in roots of teeth, which had been pivoted for seven years.

Dr. WATT said that if the pulp was perfectly healthy it needed no treatment. If diseased, it might be benefited by proper remedial agents, just as other soft tissues. When the constitution was good, and the exposure but slight and recent, if he had the control of the patient, he would expect the pulp to be protected by a deposit of osseous matter, just as he would expect osseous union, in a case of compound fracture. He had expressed these sentiments before, and had no reason to retract them. He referred to a case of exposed pulp which he had treated with astringents, and filled over it with gutta percha. In two or three months the pulp was protected by a bony deposit, and the cavity was filled with gold. About a month ago one wall of the cavity was broken away, and it became necessary to refill. While excavating, the pulp was again exposed, and bled freely. An astringent was applied, and the cavity again filled with gutta percha. The tooth was comfortable a few days ago; and it is *expected* that the pulp will be reprotected by bony matter.

Dr. FOOTE said that when covered up in this way, there was no chance for a slough to escape; and he objected to the use of escharotics and leaving decomposing animal matter in the cavity. The condition of things in a tooth was very different from that in the general system when there was an early outlet to the surface.

Dr. WATT thought it was well known that the combinations of creosote and tannin with albuminous substances, were not "decomposing animal matter."

Dr. BAXTER preferred tin foil to gold, for filling over exposed pulps.

Dr. ATKINSON was sorry to hear the remark made awhile ago, that we have too much theory. There might be too much *speculation;* but of *theory*, we were far too scarce. Correct theory was, in its very nature, a safe guide in practice. His theory, verified by practice, was that a diseased pulp might be restored. He remarked there are different degrees of inflammation. When it had not gone beyond the adhesive stage, it required no treatment. If properly prepared, success would be the uniform result in such cases. In a stage just beyond this the pulp would be painful from congestion or irritation. Again, we would find it with the circulation almost choked off at the end of the fang, when the toothache will be violent. By applying creosote, and bleeding the pulp, the pain will be relieved. The vessels are now cut, and the question is, shall we seal them up, or leave nature to do it? He would let the pulp "drain;" and if, from capillary hemorrhage, a little dead matter is left, nature knows how to dispose of it. In the first and second conditions, exposed pulps might be saved under ordinary circumstances; in the last, they could not always, nor even generally. As to the treatment of diseased pulps, he had saved a tooth alive, after removing a plug and taking away a drop of pus from under it. He was aware they would call that "jumping a high fence, but he had jumped it." He removed an amalgam plug, inserted by a neighbor, which had been worn two years and a half. He found a gutta percha cap in the bottom of the cavity; and on raising it, he saw the pulp "alive and pulsating." He referred to the case of a man with naturally good teeth, with a superior canine decayed. In excavating he exposed the pulp, producing free hemorrhage, without causing pain. Neither the instrument, nor the pressure of cotton, caused any uneasiness. He applied creosote and a horn cap, and filled. Eighteen months afterward the pulp was dead. His theory was that no nerve accompanied that vessel which he wounded; and the system, consequently, did not know that it needed matter for repair.

Dr. Foote said that nature can do her work better than we can. He thought he had been misunderstood. He would let the nerve alone, as to treatment, cover it with a nonconductor, and let nature heat it. Tole causum, he would remove the cause. Would not object to general, but would to local treatment.

Dr. TAYLOR said he wanted to get back to first principles. He agreed with the President, (Dr. Atkinson), as to the conditions defined. He would inquire of Dr. Foote, if he would leave an ulcer on the finger to nature. Dr. F. said he would not apply escharotics to an ulcer there or any place else.

Dr. ATKINSON thought Dr. F. had not treated many cases of primary syphilis.

Dr. FOOTE said he had treated a considerable number.

[Drs. A. and F. were formerly general practitioners.—REP.]

Dr. TAYLOR, resuming, said that if the exposed surface of the pulp was in a state of ulceration, the tooth could not be filled without treatment; and he was aware of no physiological or pathological reasons why an ulcer might not heal here as well as in other soft tissues.

Dr. BAXTER had used quinine, as a local application to diseased pulps, with good results.

Dr. McCLELLAND thought there was an analogy between the treatment of exposed pulps and that of the canals, or rather that portion of the nerve at the apex.

Dr. FOOTE said he rose to an explanation. He did not try to save a pulp when ulcerated.

Dr. TAFT said that when the exposure is recent, the orifice small, the constitution good, and no local irritation present, he would certainly expect to save the pulp alive. In some cases, however, even when the exposure is recent, and there is no inflammation, difficulty will arise, notwithstanding the pulp may be protected by a nonconductor. He had tried the application of nitric acid to the exposed point; and when this is done, the tooth can be filled with gold, as if no exposure had occurred. He had tested this practice for the last fifteen months, and was satisfied with its success. He had teeth die, sometimes, by the old plan; but had lost none that he knew of by this, when the circumstances were at all reasonable. It would sometimes do to leave the case to nature, after neutralizing, or removing the irritating agent; but, in many cases, this would not answer. Medication is sometimes demanded; and medicines would act here as in other cases, or as on other soft tissues. There might be a discharge from a diseased surface capable of keeping up irritation, by the arrest of which, the health of the part would be promoted. The circulation, he said, could be increased, and all the vital forces could be aroused in the pulp, as in other parts.

Dr. RICHARDSON asked if he had ever seen granulations on the surface of an exposed pulp.

Dr. TAFT, resuming, said he had seen purulent discharges from the surface of the pulp, and had seen such surface restored to health. He might judge for himself whether or not granulations had taken place.

Dr. FOOTE said he would agree that he would treat an ulcer of the pulp as an ulcer on another part—would not use caustics, but rely on cleanliness and general treatment. He would like to know how we are to distinguish a tooth that will get well without treatment from one that will not.

### III. FILLING TEETH.

Dr. FOOTE recommended the use of spunk, for drying cavities and for surrounding the tooth while filling. He said it was a good absorbent; and, as it is blackened by the absorption of moisture, it is easy to tell when the fluids of the mouth have nearly reached the cavity.

Dr. ATKINSON remarked that the public appreciates the operation of filling better now than formerly, because it is better performed and because we know better how to perform it. Every filling ought to be a unit. To accomplish this, the gold should be adhesive. As to manipulation, his "darling method" was to condense with the mallet. The desired condensation, he said, could be attained more perfectly, quicker, and with less pain to the patient than by the ordinary mode. He was sorry to say that but few understood fang filling. To do good work the operator must be able to see into the fang cavity. No difficulty need be experienced in getting to the end of the palatine fang of an upper molar. If a tooth still retains one third of its normal periosteal attachments, he thought its vitality could be preserved by judicious treatment. He referred to a case in which a young dentist applied arsenious acid to the pulp, and filled over it with amalgam cement. Abscess, both on the palatine and buccal fangs resulted. The patient wished to have the tooth extracted, but being a first molar and consequently one of the most important teeth, he undertook to save it. A probe would readily touch all the fangs, the substance between them being lost. He took out the amalgam, washed out the tooth with water and creosote, and with tincture of arnica, removed the arsenited pulp, kept fistula through the gum open, cleansed out the canals carefully, and treated them with creosote, and, when the tooth was no longer painful under pressure, he filled it. He kept the fistula open till the discharge became limpid, when he allowed it to heal, which it did in a few days. In answer to a question, he said he would "mallet the fangs, too." He described, by diagrams on the black-board, his mode of filling an upper molar, the decay being on the posterior approximate surface, and the pulp dead. He would chip out the grinding surface with mallet and chisel, till he could get into the fangs. He would insert a wedge between the necks of the decayed tooth and the one posterior to it, to keep away the gum, and to press the teeth apart, to allow space and material for a proper finish. He would, when properly prepared, fill the fangs, and then the main cavity, restoring to the tooth as nearly its original shape as possible. This restoration of shape he regarded as very important. He prepared his gold by rolling it into loose ropes, and cutting it into pellets, or blocks; and in filling, he passes each pellet through an alcohol flame, just before putting it into the tooth. By request of Dr. Taft, he referred to the formation of new alveolar process, and described two cases, in one of which new process was formed from one canine to the other,—in the other, the gum and process were destroyed by caustic potash, and, in time, restored. The new process, he said, would not be as sharp as the natural formation. In treating a case with a view to the formation of new process, he kept away irritants, and used, locally, tincture of iodine, creosote, and tannin.

IV. IRREGULARITIES OF THE TEETH, THEIR CAUSES AND MODES OF TREATMENT.

Dr. ATKINSON thought it important to let the fangs be fully develped before the process of regulating is commenced. He described, by diagrams, his various modes of operating; but without these it would be impossible to do justice to his remarks.

[The further discussion of this subject was somewhat conversational,—cases and appliances being exhibited and discussed with freedom. All seemed both instructed and delighted. This, the most interesting part of the proceedings, can not be put on paper. We wish our readers could have been all present.—REP.]

V. STRUCTURE AND NUTRITION OF THE DENTAL TISSUES.

This subject was laid over, by a unanimous vote, for consideration at the next annual meeting.

VI. HEMORRHAGE FOLLOWING THE EXTRACTION OF TEETH, AND THE BEST TREATMENT.

Dr. MCCLELLAND referred to a case of hemorrhage after the extraction of a lower wisdom tooth. He used pressure and the sesquichloride of iron, with success. He left the pledget in, till it came away of itself. Another case, a young man, bled six hours. He succeeded by the same treatment. He relied on pressure, and the sesquichloride, which combines with the blood. He would advise hemorrhagic patients to avoid exercise. Before he adopted the sesquichloride, he used turpentine and tannin as a styptic.

Dr. Driggs said his treatment was much like Dr. McClelland's, except that he seldom used astringents. His main reliance was on pressure by packing the cavity.

Dr. Watt said the treatmont of hemorrhage may be either local or constitutional, or both. Many practitioners, he said, had indefinite ideas as to the action of styptics. A true styptic would combine with some constituent of the blood and form a solid compound insoluable in the blood. Hence, nitrate of silver, and kindred salts, though often used as such, were not worthy to be ranked as styptics. The sesquichloride of iron, or, as it is often called, the perchloride, is a powerful styptic, and so is the persulphate of iron. Tannin, he said, was also very reliable. Any one of these, aided by pressure properly applied, would afford us all we could expect from local treatment. Acetate of lead and opium would often prove useful in treating the constitution. When it is known that the patient is predisposed to hemorrhage, the constitution should be treated before the tooth is extracted.

Dr. Richardson said the effect of local treatment would depend very much on the state of the blood, as to quantity and quality. If the patient were plethoric he would deplete. As to pressure, it could sometimes be best applied by the adaptation of a plate to the parts. He referred to a case, in which he was convinced that the hemorrhage was vicarious; and he referred it to a physician.

[Here the regular discussions ended; but the members still lingered and continued to exchange ideas on various practical subjects. We hope many will be present to hear and take part in the discussions of No. 5, next year, as well as to aid in and enjoy all the proceedings of this pioneer Association.] W.

